# Supplementary material for: Ongoing uncoordinated anthropogenic emission abatement promotes atmospheric new particle growth in a Chinese megacity
Source: Nat Commun. 2025 Jul 21;16:6720. doi: 10.1038/s41467-025-62011-6 (PMC12280209; doi:10.1038/s41467-025-62011-6)
Supplement: Supplementary file 1 — Supplementary Information [file 41467_2025_62011_MOESM1_ESM.pdf]

Supporting Information for

Ongoing uncoordinated anthropogenic emission abatement promotes atmospheric new particle growth in a Chinese megacity

Lizi Tang[1#], Zeyu Feng[1#], Dongjie Shang[1], Linghan Zeng[1], Zhijun Wu[1,2], Hui Wang[3], Shiyi Chen[1], Xin Li[1,2], Limin Zeng[1,2], Jianlin Hu[2], Min Hu[1,2*]

[1]State Key Laboratory of Regional Environment and Sustainability, International Joint Laboratory of Regional Pollution Control, Ministry of Education (IJRC), College of Environmental International Joint Laboratory for Regional Pollution Control, Ministry of Education (IJRC), Sciences and Engineering, Peking University, Beijing 100871, China

[2]Collaborative Innovation Center of Atmospheric Environment and Equipment Technology, Nanjing University of Information Science & Technology, Nanjing 210044, China

[3]Institute of Climate and Energy systems, Troposphere, ICE-3, Forschungszentrum Jülich, 52425 Jülich, Germany

# These authors contributed equally: Lizi Tang and Zeyu Feng

*Corresponding author: Min Hu (minhu@pku.edu.cn)

This PDF file includes:

Supplementary Methods 1 to 5

Supplementary Figures 1 to 28

Supplementary Tables 1 to 3

Supplementary References

Supplementary Method 1. Parameterization of new particle formation (NPF) and growth rate (GR)

In this study, a typical NPF event was characterized by a significant increase in $PN_{1.5\text{-}3}$ (particle number concentration in the size range of 1.5-3 nm) to > 20,000 cm^{-3}, or a significant increase in $PN_{3\text{-}10}$ (when $PN_{1.5\text{-}3}$ data is lacking) to > 4000 cm^{-3}, lasting for more than 2 h.[1] The days without particle formation were defined as non-event days. Other days that failed fulfilling the criteria to be classified as either NPF event or non-event days were regarded as undefined days.

The growth rate (GR) was obtained by the mode-fitting method[2]. The particle number size distribution (PNSD) during NPF event days were fitted as the sum of three-mode lognormal distribution. GR was calculated as the variation of the geometric mean diameter D_m of newly formed mode in unit internal[2], The growth rates in three particle size ranges, $GR_{1.5\text{-}3}$, $GR_{3\text{–}15}$, and $GR_{15\text{–}25}$ were calculated separately, where the subscripts indicate the size range:

$$\mathrm{GR} = \frac{\Delta D_m}{\Delta t}$$

(1)

To evaluate the scavenging effects of preexisting particles on condensable vapors, the condensation sink (CS) was calculated as follow[2]:

$$\mathrm{CS} = 2\pi \mathrm{D} \sum \beta_m\left(D_{p,i}\right) D_{p,i} N_i$$

(2)

where D is the diffusion coefficient of the condensing vapor (here we use sulfuric acid as the representing vapor), β_m is the transition regime correction factor, and $D_{p,i}$ and N_i are the diameter and number concentration of particles in the size bin i, respectively.

Supplementary Method 2. Supplementary measurement instruments

Particle number size distribution (PNSD) of 1.5-3 nm particles was measured with a nano condensation nucleus counter system (nCNC) consisting of a particle size magnifier (PSM, Model A10, Airmodus Inc., Finland) and a butanol condensation particle counter (CPC, Model A20, Airmodus Inc., Finland). PNSD in the size range of 3-698 nm was obtained by integrating two sets of scanning mobility particle spectrometers (SMPS). The first SMPS measures particles with sizes between 3 and 45 nm, consisting of a TSI Model 3085 DMA and a TSI Model 3776 CPC. The second SMPS measures particles between 45 and 698 nm, consisting of a TSI Model 3081 DMA and a TSI Model 3776 CPC. Detailed procedure can be found in previous study.[3] Tracer gaseous pollutants were continuously detected by a series of online monitoring system manufactured by Thermo Electron Corporation (O_3 (Model 49i), NO-NO_2-NO_x (Model 42i) and SO_2 (Model 43i-TLE). Meteorological parameters including wind speed (WS), wind direction (WD), temperature (T) and relative humidity (RH) were measured by the automatic meteorological station (Met one Instrument Inc). The photolysis frequencies of O_3 (JO^1D) were monitored by a spectroradiometer, following the procedure described by Wang, et

al.[4]. 99 types of volatile organic compounds (VOCs) were measured by the online gas chromatography and mass spectrometry (GC-MS) system[5]. The AVOC species used for the analysis in this study are shown in Supplementary Table 3. Due to maintenance work on GC-MS system, the AVOC data is relatively limited. In contrast, the NO_x data is more complete. Our analysis indicated that the f_{con}-NO_x relationships for days with available AVOC data are largely consistent with those derived from all measurement days (Supplementary Fig. S25). It suggested that the key findings are robust although the AVOC and NO_x datapoints do not fully align (Fig. 3a and 3b).

Supplementary Method 3. Workflow for retrieving OOM sources

Firstly, the fluorinated contaminants and nitrated phenols are distinguished according to the unique fingerprint molecules from the literature and excluded from the analysis, considering that nitrated phenols are too volatile to have significant contribution to particle growth[6,7]. Isoprene OOMs are also distinguished from fingerprint molecules. Then, the remaining molecules are classified with the carbon number (nC), the nitrogen number (nN), the double bond equivalence (DBE = (2 × nC + 2 – nH - nN) / 2) and the effective oxygen number (nO_{eff} = nO-2×nN). The criteria of nC = 10, $nO_{eff} \geq 4$, and $2 \leq DBE \leq 4$ is used for monoterpene OOMs identification based on their reported composition[8,9]. The molecules with DBE ≥ 3 are recognized as aromatic OOMs, while the molecules with DBE ≤ 1 are treated as aliphatic OOMs. For the molecules with DBE = 2, if $nO_{eff} \geq 6$, they could be classified as aromatic OOMs; if $nO_{eff} < 5$ and nN ≤ 1, they would be assigned to aliphatic OOMs. The molecules with $nO_{eff} = 5$ or $nO_{eff} < 5$ and nN > 1 cannot be distinguished between aromatic and aliphatic origins, and are termed as undistinguished OOMs. The workflow has its limitations and uncertainties as the current understanding of gaseous OOM formation mechanisms is not comprehensive. Nie, et al.[6] and Yang, et al.[10] elucidated the anticipated uncertainties and constraints of this workflow, and tested the performance of this workflow using the known OOM peak lists from various well-controlled laboratory studies. The results revealed accuracy exceeding 95% for the tested aromatic OOMs, nearly 100% for OOMs originating from n-decane, and approximately 75% for OOMs derived from cyclohexane. Therefore, the workflow performs well and is applicable to this study. We refer to the classified products as Aro-OOMs, Ali-OOMs, MT-OOMs and IP-OOMs, respectively. The OOMs cannot be distinguished between aromatic and aliphatic origins are referred as Undis.

Supplementary Method 4. Vapor condensation growth modeling

The net condensation flux is calculated by:

$$\frac{dc_{i,p}}{dt} = N_p \cdot \sigma_{i,p} \cdot k_{i,p} \cdot F_{i,p}$$

(3)

where N_p is the particle number concentration at a given size d_p, $\sigma_{i,p}$ is the particle-vapor collision cross-section between vapor in VBS bin i and a given particle size d_p, $k_{i,p}$ is the

deposition rate of OOM vapor at the particle surface, and $F_{i,p}$ is the driving force of condensation of vapor i. $\sigma_{i,p}$ is obtained as:

$$\sigma_{i,p} = \frac{\pi}{4}(d_p + d_i)^2$$

(4)

where d_p and d_i are the diameter of particle and condensable vapor.

The deposition rate $k_{i,p}$ depends on the mass accommodation coefficient $\alpha_{i,p}$, the center mass velocity of particle and vapor $v_{i,p}$, and the non-continuum dynamic factor $\beta_{i,p}$:

$$k_{i,p} = \alpha_{i,p} v_{i,p} \beta_{i,p}$$

(5)

Here, $\alpha_{i,p}$ is assumed to be 1. $v_{i,p} = \sqrt{\frac{8RT}{\pi u_{i,p}}}$, and $u_{i,p} = \frac{M_i M_p}{M_i + M_p}$, where M_p and M_i are the molar mass of organic vapors and particles. $\beta_{i,p} = \frac{Kn \cdot (Kn+1)}{Kn^2 + Kn + 0.283 Kn \cdot \alpha_{i,p} + 0.75\alpha_{i,p}}$ is the adjusted Fuchs & Sutugin correction factor. The Knudsen number $Kn = \frac{2\lambda}{d_i + d_p}$. The mean free path $\lambda = 3(D_i + D_i)(\bar{c_i}^2 + \bar{c_p}^2)^{-\frac{1}{2}}$, which is adjusted for the non-negligible effect of vapor-molecular size. $D_{i/p} = k_B T C_C(d_{i/p})/(3\pi\eta d_{i/p})$, which is the diffusion coefficient of the vapor or particle with the Cunningham slip correction $C_C(d_{i/p}) = 1.0 + Kn(1.165 + 0.483 \cdot \exp(-0.997/Kn)$. The mean thermal velocity $\bar{c}_{i/p} = \sqrt{8RT/\pi d_{i/p}}$.

$$F_{i,p} = c_{i,g} - a_{i,p} c_i^0$$

(6)

where $c_{i,g}$ and c_i^0 are the vapor concentration in gas phase and saturation vapor concentration, respectively, and $a_{i,p}$ is the particle phase activity. $a_{i,p} = X_{i,p}\gamma_{i,p}K_{i,p}$, where $\gamma_{i,p}$ is the mass-based activity coefficient in the condensed phase, which is assumed to be 1. $K_{i,p}$ is the Kelvin coefficient, and is estimated by $K_{i,p} = 10^{\frac{d_k}{d_p}}$, where d_k is the Kelvin-diameter and is assumed to be 4.8 nm. $X_{i,p} = c_{i,p}/c_p$ is the mass fraction of vapor i in particle phase. $c_{i,p}$ is the mass of vapor i in particle phase, and c_p is the sum of $c_{i,p}$ for all the condensed vapors in particle phase.

The simulation starts with the occurrence of NPF events, and the initial particle diameter is considered as the fitted diameter (1.3-2 nm) when NPF events start to occur. The initial particulate mass of the seed is assumed to be non-volatile and doesn't change during the simulation. The initial $c_{i,p}$ of newly formed particles are negligible. Then in each time step (10 min), the condensation flux of vapor i is calculated by the dynamic equation (3). By multiplying the condensation flux by the time interval, $c_{i,p}$ and c_p can be obtained. We assume that the particulate matter is spherical, thus the simulated size of the particles in different time steps can be obtained with an assumed particle density ρ_{OA}.

Additionally, the net condensation flux can be expressed as:

$$\sum_i \frac{dc_{i,p}}{dt} = \frac{d(\rho_{OA}\frac{\pi}{6}d_p^3 N_p)}{dt} = \rho_{OA}\frac{\pi}{6}N_p 3d_{i,p}^2\frac{dd_p}{dt}$$

(7)

The growth rate at d_p can be calculated as:

$$GR = \frac{dd_p}{dt} = \sum_i \frac{dc_{i,p}}{dt} \cdot \frac{2}{\rho_{OA}\pi N_p d_p^2}$$
$$= \sum_i N_p \cdot \frac{\pi}{4}\left(d_p + d_i\right)^2 \cdot \alpha_{i,p} v_{i,p} \beta_{i,p} \cdot (c_{i,g} - a_{i,p} c_i^0) \cdot \frac{2}{\rho_{OA}\pi N_p d_p^2}$$
$$= \sum_i \frac{1}{2}\frac{\left(d_p + d_i\right)^2}{\rho_{OA} d_p^2} \cdot \alpha_{i,p} v_{i,p} \beta_{i,p} \cdot (c_{i,g} - a_{i,p} c_i^0)$$

(8)

Supplementary Method 5. Analysis for the impact of coagulation effect on the observed GR

The observed GR was apparent GR calculated using the mode-fitting (FT) method or appearance time (AT) method, which also include intramodal and extramodal coagulation contribution, apart from vapor condensation. To eliminate the impact of coagulation on apparent GR, the simplified aerosol general dynamic equation (GDE) method was adopted to obtain GR contributed solely by vapor condensation, as introduced by Yu, et al.[11]. It was found GR from GDE method (GR_{GDE}) is comparable to or a little bit higher than that from AT and FT method in 1-25 nm, demonstrating the dominant role of vapor condensation in new particle growth (Fig. S26). The relatively higher GR_{GDE} than apparent method may come from the uncertainties in PNSD measurements and GR calculation. Thus, the simulated GR can be directly compared with the apparent GR, to evaluate the contribution of observed OOMs and sulfuric acid to new particle growth. The ratio of simulated GR to GR_{GDE} is displayed in Fig. S27.

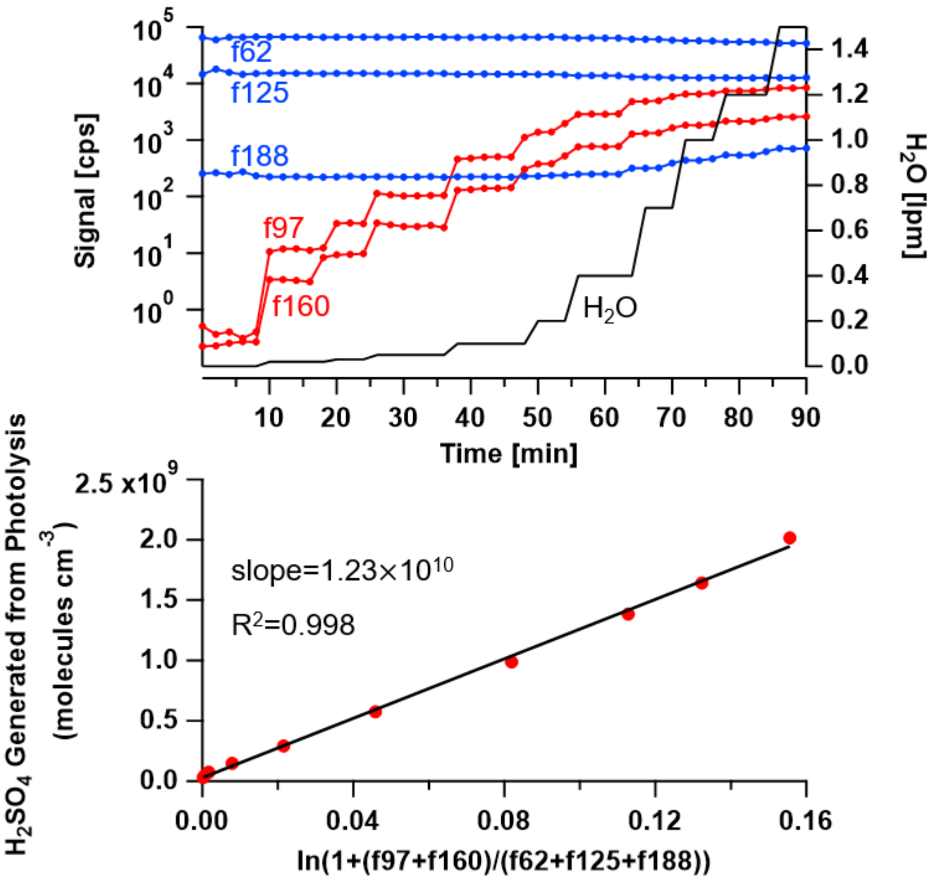

Supplementary Fig. 1. Results of the H_2SO_4 calibration experiment for Nitrate-CI-APi-TOF during the observation in autumn 2021 in Beijing. Time series of signals and flow rate of H_2O are shown in the upper panel. The calibration curve retrieved from the above time series is shown in the lower panel. The information of calibration experiment can be found in Kürten, et al.[12]. H_2SO_4 is produced from the reaction of SO_2 and OH radicals, which is produced in situ through the UV photolysis of H_2O. Therefore, different flow rate of H_2O (H_2O [lpm]) correspond to different H_2SO_4 concentration. f62, f125, f188, f97 and f160 are the signal of NO_3^-, $HNO_3 \cdot NO_3^-$, $(HNO_3)_2 \cdot NO_3^-$, HSO_4^- and $HSO_4^- \cdot HNO_3$, respectively.

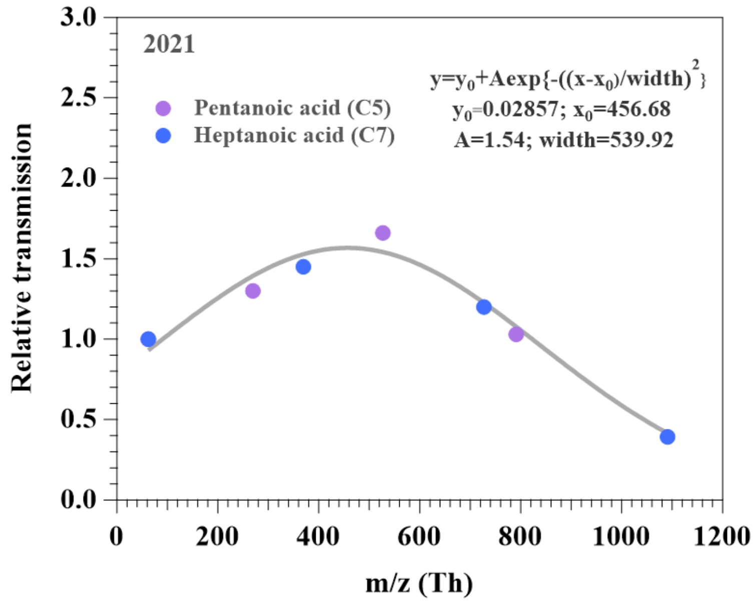

Supplementary Fig. 2. Relative transmission efficiency curve during the observation in autumn 2021 in Beijing

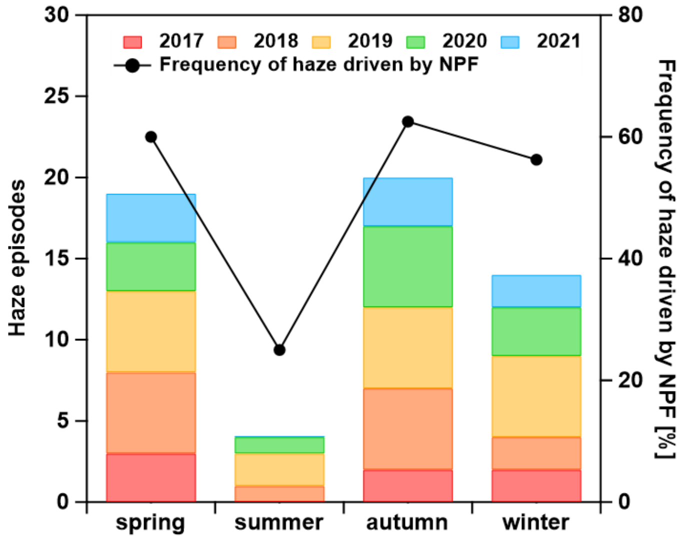

Supplementary Fig. 3. Haze episodes and frequency of haze by NPF in different season from 2017 to 2021. A haze episode is defined as a continuous period with daily average $PM_{2.5}$ concentrations exceeding 75 μg m^{-3} for two or more consecutive days. The haze driven by NPF is defined by the observation of NPF events within the 7-day period preceding a haze episode, accompanied by a sustained increase in particulate matter mass from the onset of NPF until the haze pollution.

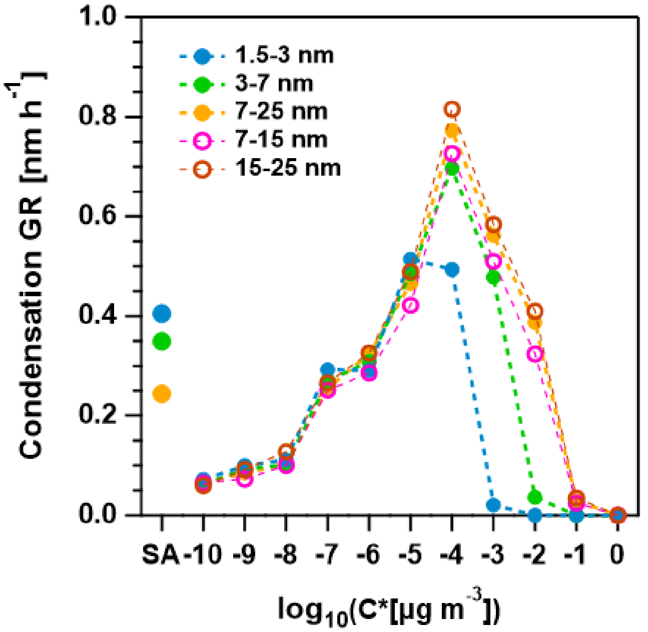

Supplementary Fig. 4. The condensation GR contributed by OOMs with different volatility for 1.5-25 nm particles during the observation in autumn 2021 in Beijing. The volatility distribution of condensation GR in 7-15 nm and 15-25 nm are similar with that in 7-25 nm.

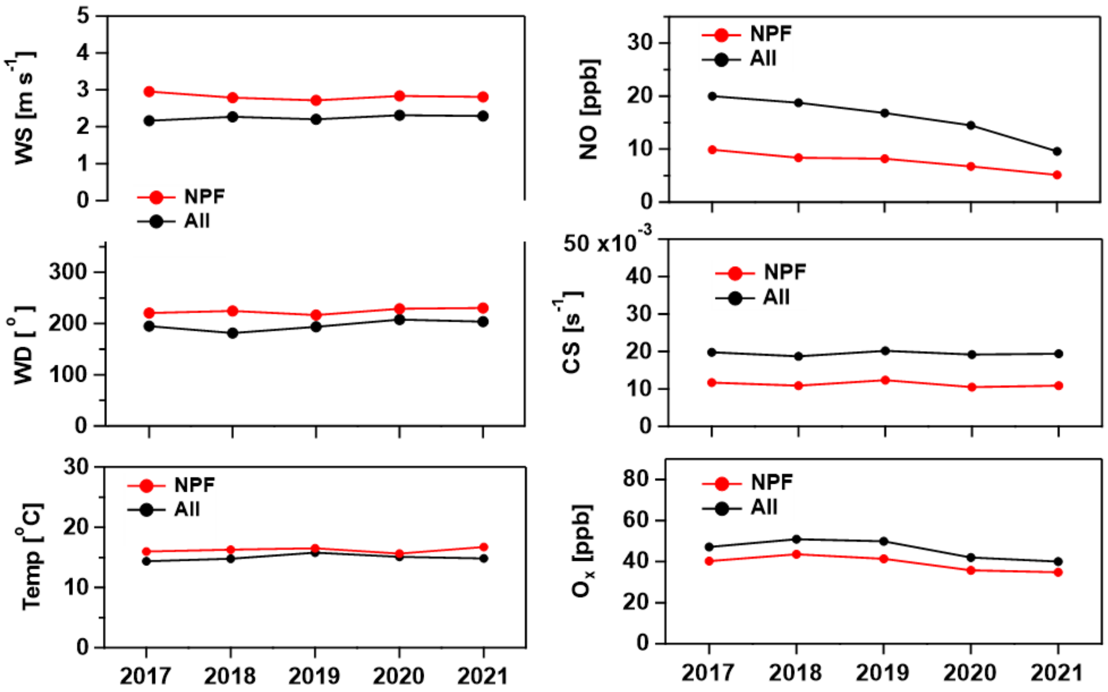

Supplementary Fig. 5. Variations in average wind direction (WD), wind speed (WS), temperature, NO, condensational sink (CS) and O_x (NO_2 + O_3) in autumn from 2017 to 2021 in Beijing.

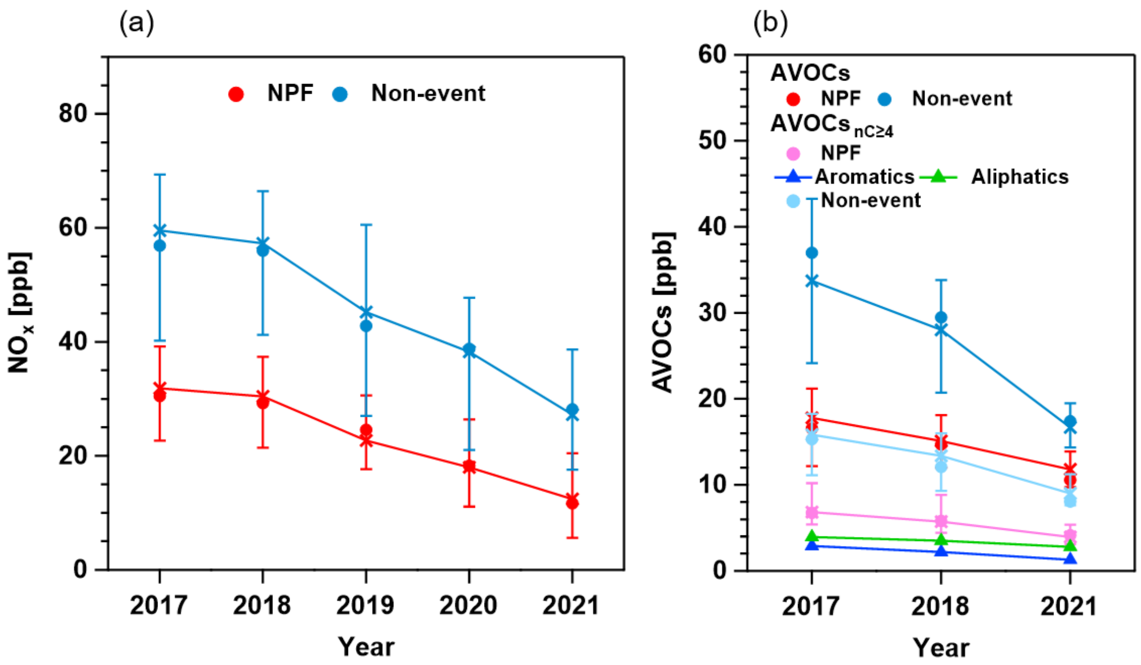

Supplementary Fig. 6. Variations of NO_x and AVOCs on NPF days and non-event days in autumn from 2017 to 2021. (a) Variations of NO_x on NPF days and non-event days. (b) Variations of AVOCs, AVOCs with nC ≥ 4 as well as aromatic and aliphatic VOCs in NPF days and non-event days in autumn 2017, 2018 and 2021. The whiskers correspond to the 25th and 75th percentiles. The circular and cross markers represent the median and mean values, respectively.

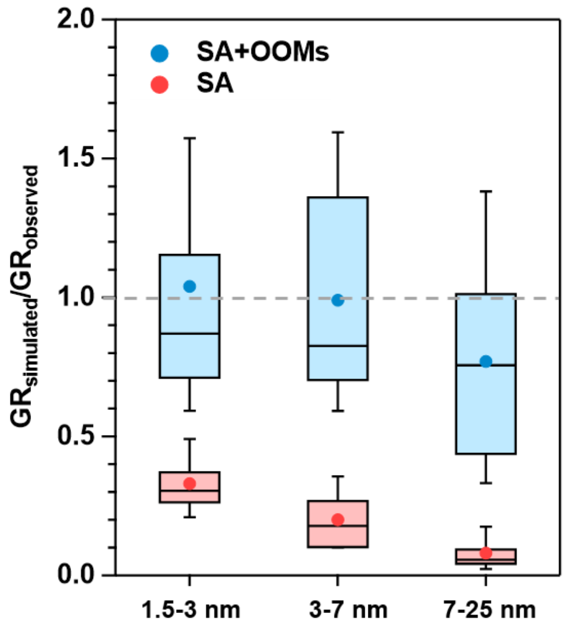

Supplementary Fig. 7. The ratio of simulated GR to observed GR (mode-fitting method) for different particle size bins during the observation in autumn 2021 in Beijing. The simulated GR due to sulfuric acid condensation is shown in red, and the blue ones are GR from sulfuric acid plus OOMs condensation. The whiskers are the 90th and 10th percentiles; the upper and lower boundaries of the boxes indicate the 75 and 25th percentiles; and the lines and markers are the median and mean values, respectively.

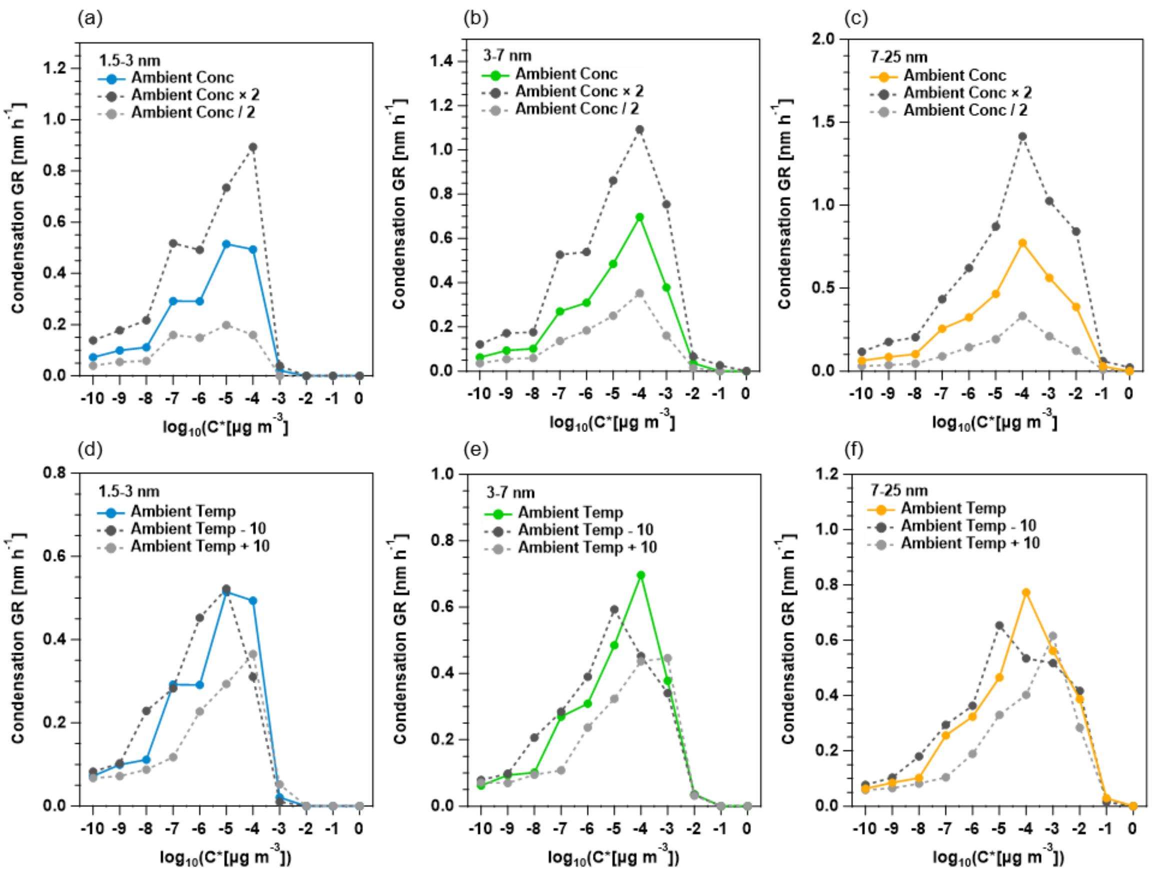

Supplementary Fig. 8. The sensitivity analysis of condensation GR to (a-c) condensable OOM concentration and (d-f) temperature for 1.5-3 nm, 3-7 nm and 7-25 nm particles during the observation in autumn 2021 in Beijing. Specifically, the OOM concentrations were scaled by a factor of 2 and temperatures were perturbed by ± 10 K respectively.

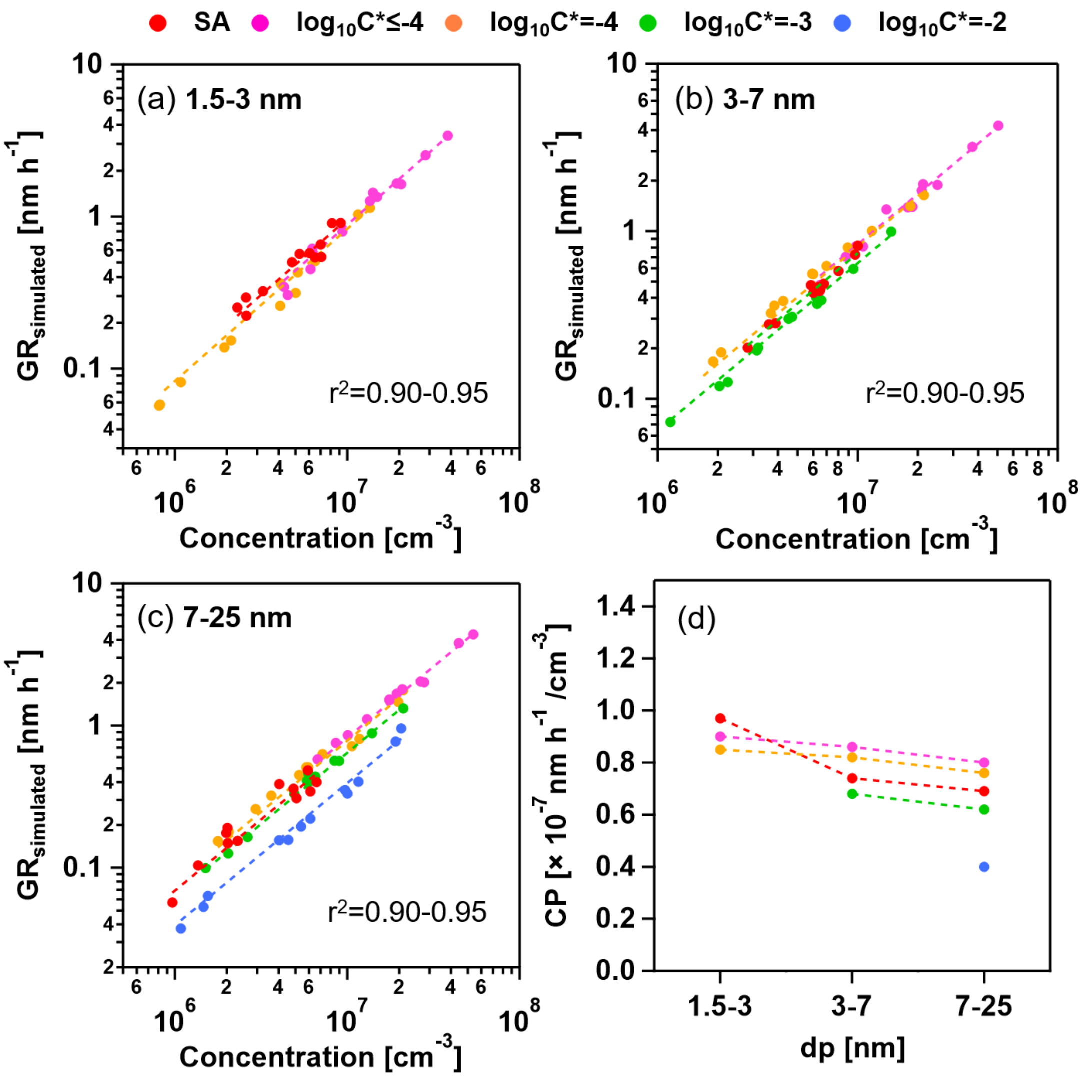

Supplementary Fig. 9. Condensation potential of condensable vapors with different volatility during the observation in autumn 2021 in Beijing based on average vapor concentrations during the new particle growth period. Relationships between simulated condensation GR and average vapor concentrations during the new particle growth period for (a) 1.5-3 nm, (b) 3-7 nm, and (c) 7-25 nm particles. The vapors include sulfuric acid, OOMs with $\log_{10}C^* \leq -4$, $\log_{10}C^* = -4$, -3, and -2. Dashed lines are the linear regressions for different vapors. (d) Slopes of linear regressions ($GR_{simulated}$ vs concentration), i.e., condensation potential (CP), for different vapors in different particle size bins.

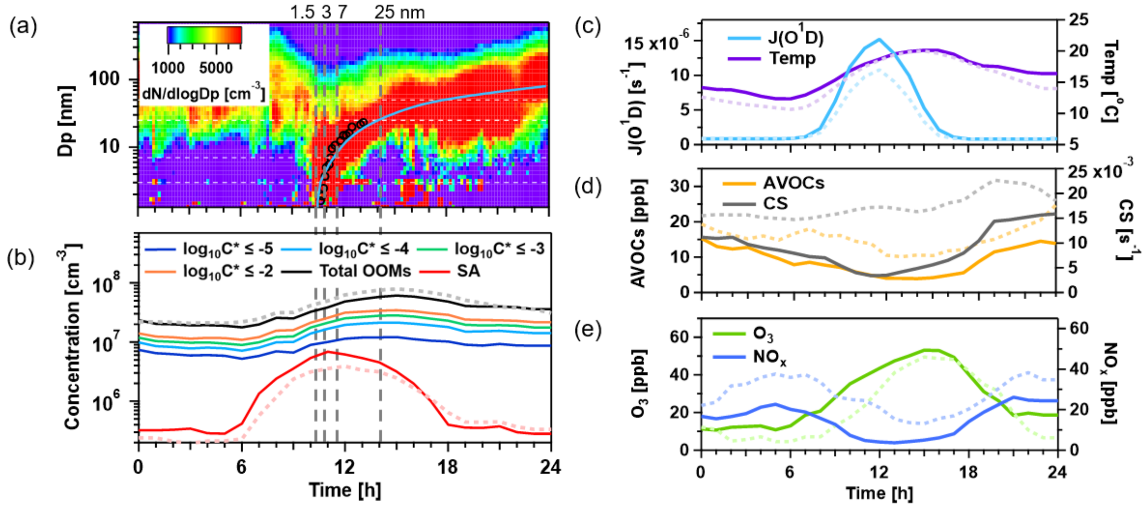

Supplementary Fig. 10. The diurnal variations of particle number concentration size distribution (PNSD), OOMs, SA and other related parameters during the observation in autumn 2021 in Beijing. Diurnal variations of (a) PNSD, (b) OOMs and SA, (c) $J(O^1D)$ and temperature, (d) AVOCs and CS, (e) O_3 and NO_x in NPF days and non-event days. The PNSD on 29 September in 2021 exhibits an example of NPF days. In (a), the black dots represent the measured particle growth route, calculated using the mode-fitting method, and the blue line represent the modeled particle growth route. In (b-e), the solid line is the average value in NPF days. The dashed line is the average value in non-event days.

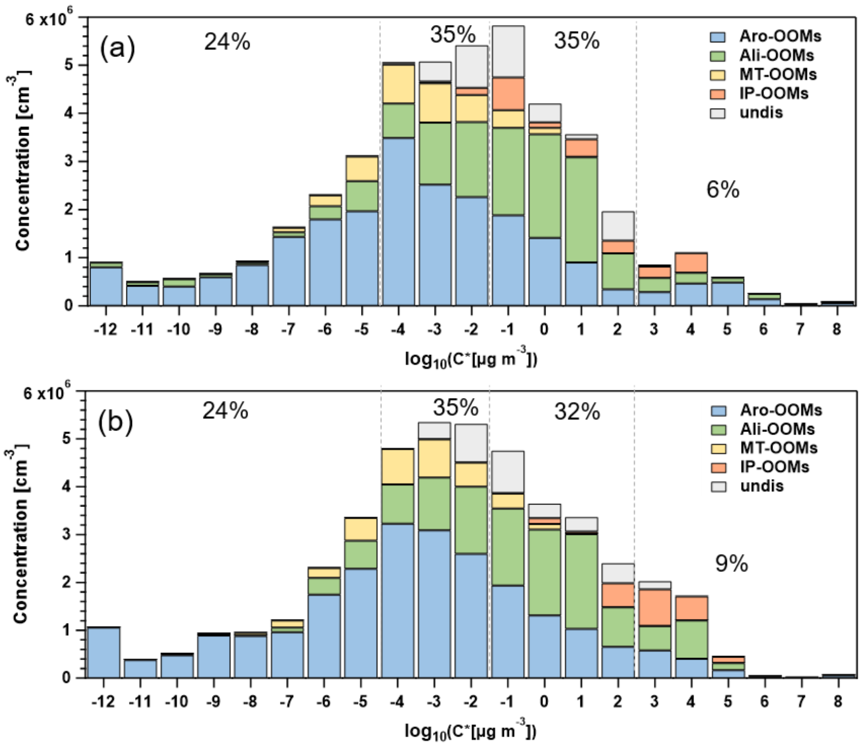

Supplementary Fig. 11. Averaged volatility distribution of OOMs from different sources (Aro-OOMs, Ali-OOMs, MT-OOMs and IP-OOMs) at measured ambient temperature in NPF days during the observation in autumn 2021 in Beijing, obtained using volatility calculation method (a) in this study and (b) in Qiao, et al.[7]. The proportions in the figure represent the percentages of OOMs in $\log_{10}C^* \leq -5$, $-4 \leq \log_{10}C^* \leq -2$, $-1 \leq \log_{10}C^* \leq 2$, and $\log_{10}C^* \geq 3$.

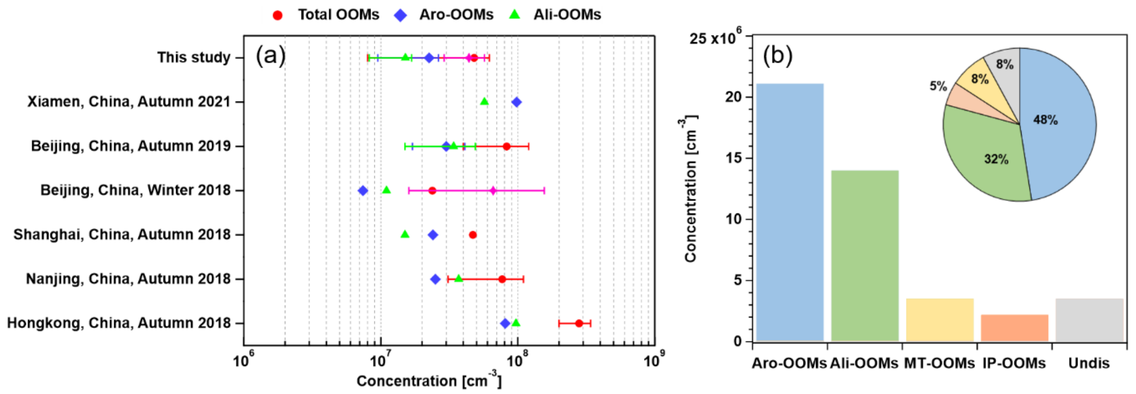

Supplementary Fig. 12. The concentration of the total OOMs and OOMs originated from different precursors. (a) Comparison of the total OOMs (red), Aro-OOMs (blue) and Ali-OOMs (green) at different locations in autumn and winter. Data for autumn 2021 in Xiamen are from Yang, et al.[10], data for autumn 2019 in Beijing are from Guo, et al.[13], data for winter 2018 in Beijing, autumn 2018 in Shanghai, Nanjing and Hong Kong are from Nie, et al.[6], and data for NPF days in autumn 2018 in Beijing are from Qiao, et al.[7]. The pink point represents the OOM concentration observed in NPF days. Points are mean concentrations, and bars on x-axis direction correspond to the 25th and 75th values. (b) Absolute concentrations and contributions of OOMs originated from different precursors in NPF days during the observation in autumn 2021 in Beijing. Aro-, Ali-, MT-, IP-OOMs and Undis stand for OOMs originated from aromatics, aliphatics, monoterpene, isoprene and undistinguished source.

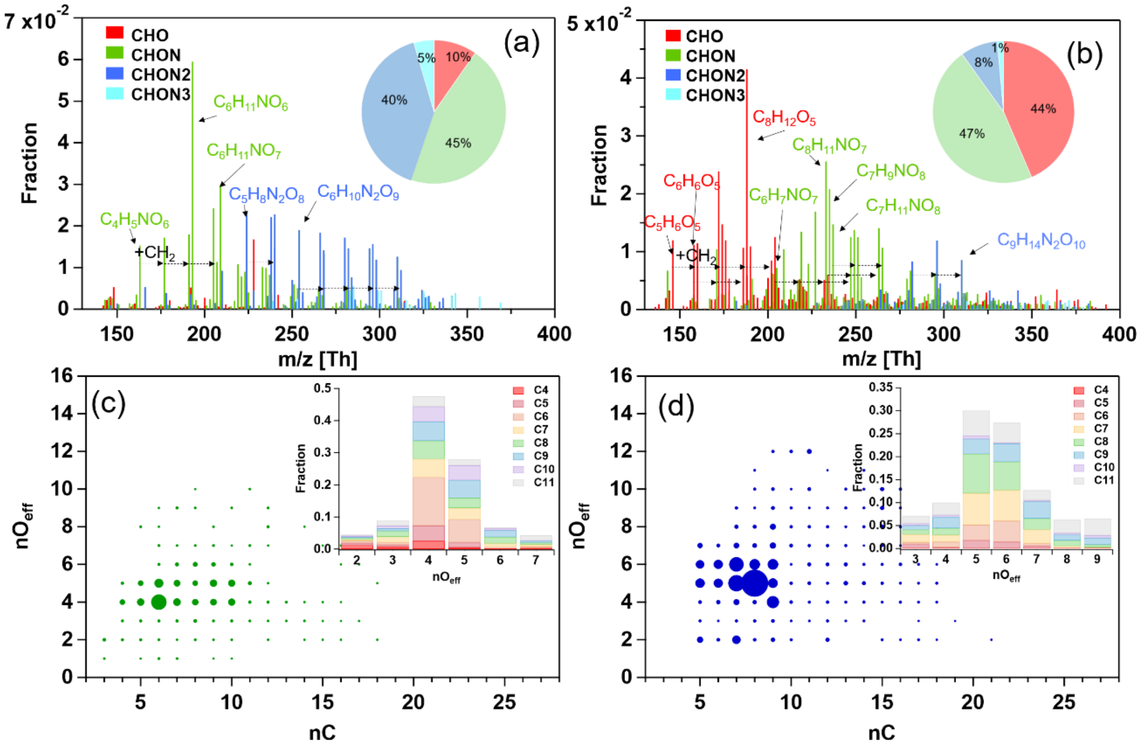

Supplementary Fig. 13. The spectra and characteristics for Ali-OOMs and Aro-OOMs during the observation in autumn 2021 in Beijing. The spectra for (a) Ali-OOMs and (b) Aro-OOMs. The color reflects the number of nitrogen atoms (nN). The sticks connected by arrows represent the homologs. The distribution of measured (c) Ali-OOMs and (d) Aro-OOMs as functions of effective oxygen number (nO_{eff}) and carbon number (nC). The symbol size is scaled by the OOM concentration. The distributions of nO_{eff} for Aro-OOMs and Ali-OOMs are present in the subplots. The color reflects the number of carbon atoms, C11 means the carbon number ≥ 11.

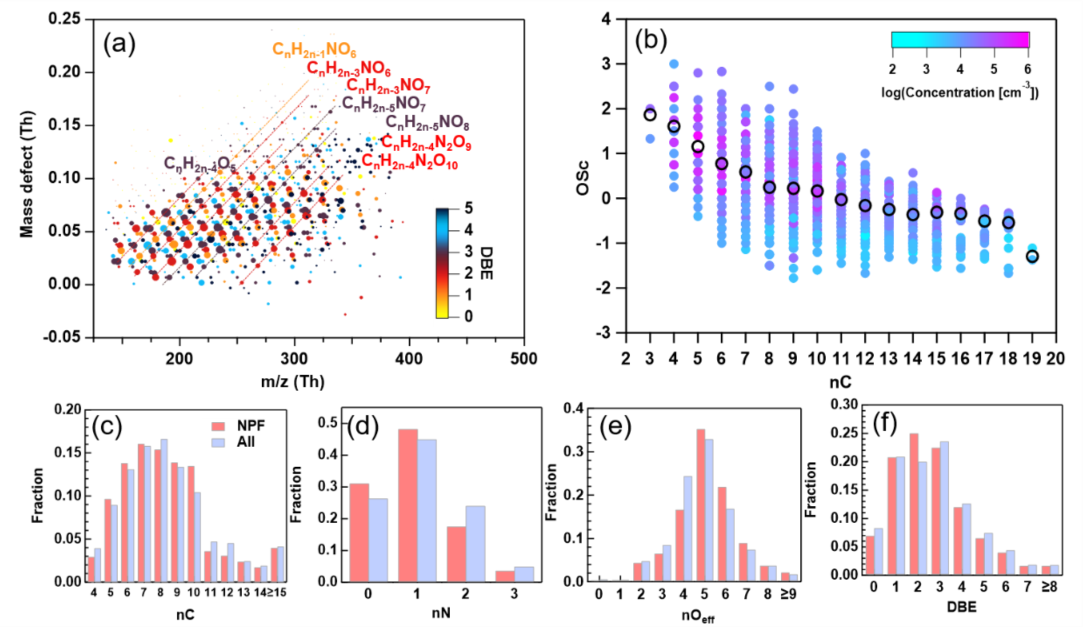

Supplementary Fig. 14. Mass defect plot and molecular characteristics of the OOMs during the observation in autumn 2021 in Beijing. (a) Mass defect plot of the OOMs identified in NPF days. The dots are colored with the double bond equivalence (DBE) and scaled by the logarithm of the OOM concentration. The dots connected by lines represent the homologs or serial products. (b) The carbon oxidation state (OSc) distribution of OOMs as a function of carbon number (nC) in NPF days. The dots are colored with the logarithm of the OOM concentration. The black circle denotes the concentration-weighted mean OSc of the measured OOM for each carbon number. (c) The number of carbon (nC), (d) effective oxygen number (nO_{eff}), (e) nitrogen (nN), and (f) double bond equivalence (DBE) distributions of the identified OOMs in NPF days (red) and the whole period (blue).

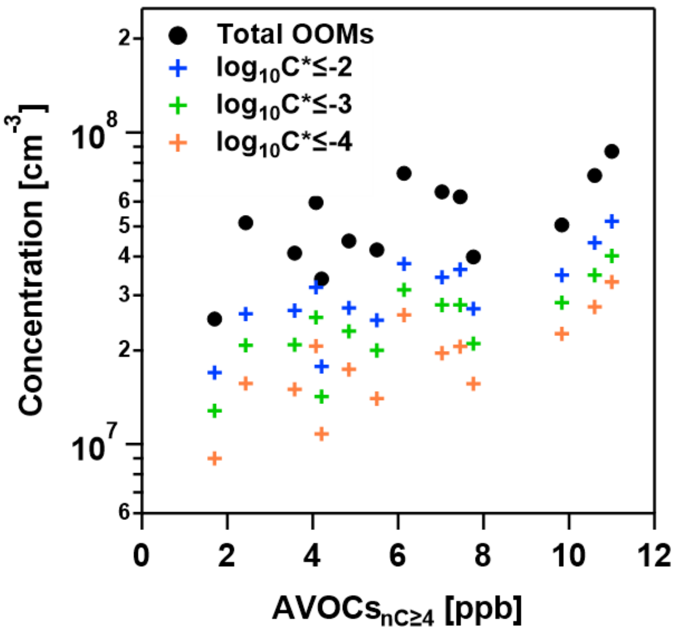

Supplementary Fig. 15. Relationships between daily average concentrations of OOMs and AVOCs$_{nC\geq4}$ at campaign-average temperature (289 K) during the observations in autumn 2021 of Beijing.

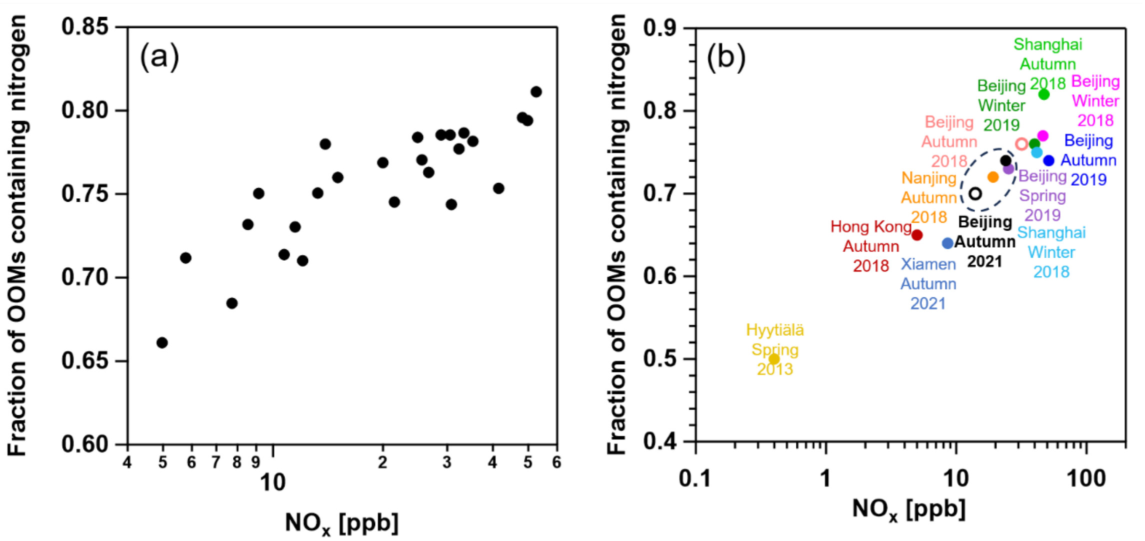

Supplementary Fig. 16. Impact of NO_x on nitrogen content of OOMs. (a) Relationships between daily average fraction of OOMs containing nitrogen and daily average NO_x concentrations during the observation in autumn 2021 in Beijing. (b) Relationships between fraction of OOMs containing nitrogen and NO_x concentration from different measurements. Data for spring, autumn and winter 2019 in Beijing are from Guo, et al.[13], data for winter 2018 in Beijing, autumn 2018 in Shanghai and Nanjing are from Nie, et al.[6], data for winter 2018 in Shanghai are from Tian, et al.[14], data for autumn 2018 in Hong Kong are from Zheng, et al.[15], data for autumn 2021 in Xiamen are from Yang, et al.[10], data for spring 2013 in Hyytiälä are from Bianchi, et al.[16], data for NPF days in autumn 2018 in Beijing are from Qiao, et al.[7]. Note that the OOM data in Xiamen is anthropogenic OOM data, which can represent the levels and characteristics of the total OOMs.

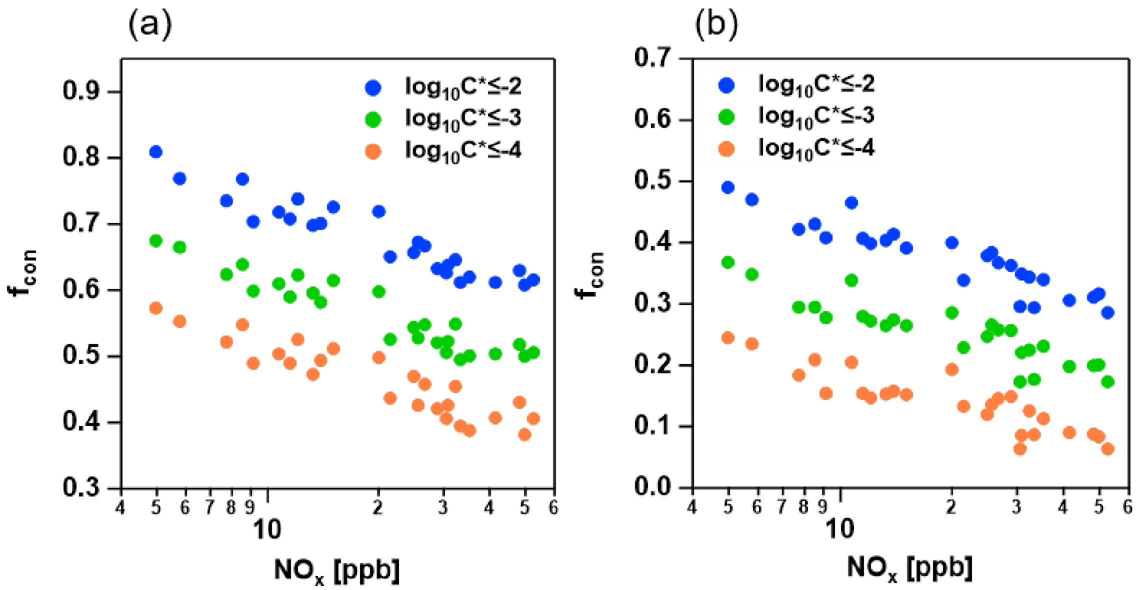

Supplementary Fig. 17. Relationships between daily average fractions of condensable OOMs (f_{con}) and daily average NO_x concentrations for (a) Aro-OOMs and (b) Ali-OOMs at campaign-average temperature (289 K) during the observation in autumn 2021 in Beijing.

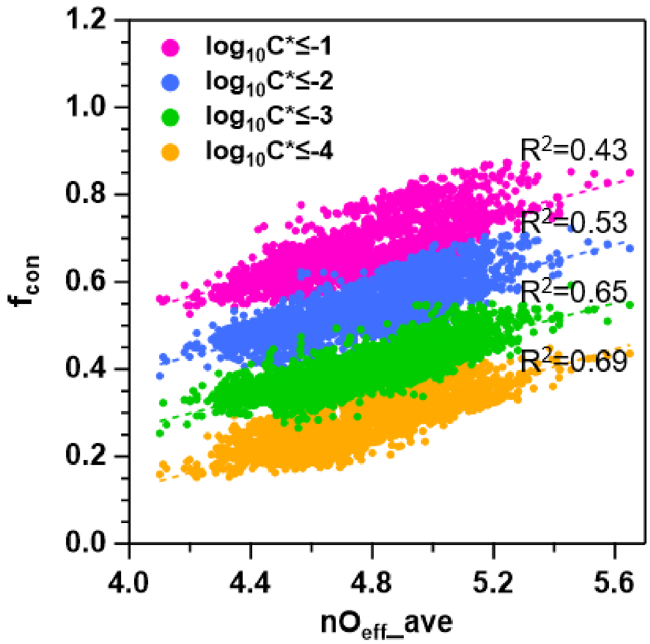

Supplementary Fig. 18. Relationships between the fraction of condensable OOMs (f_{con}) and concentration-weighted nO_{eff} of OOMs at 300 K during the observation in autumn 2021 in Beijing. The dash lines are the linear regressions of f_{con} and concentration-weighted nO_{eff} of OOMs.

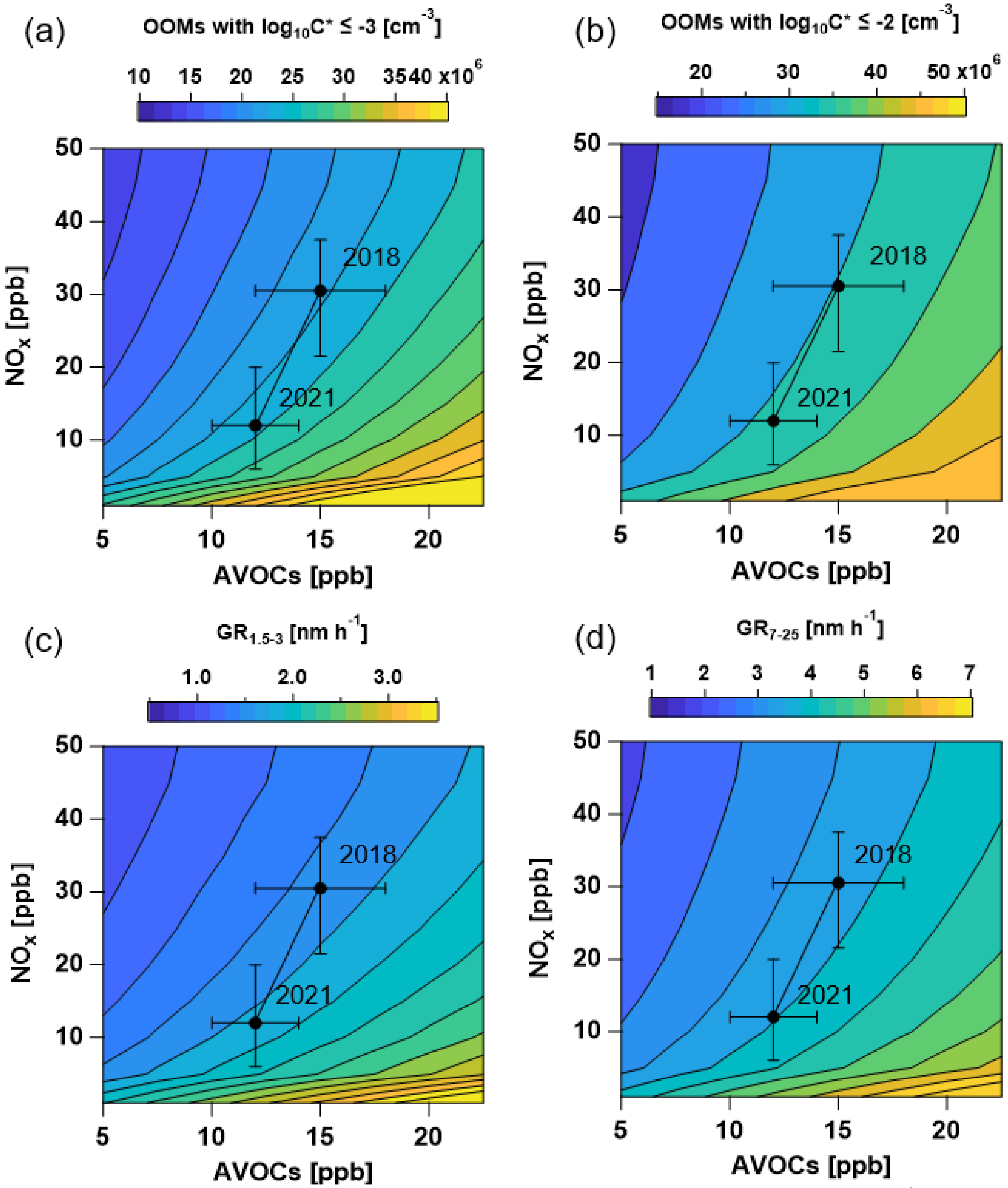

Supplementary Fig. 19. Isopleth plots for concentrations of OOMs with $\log_{10}C^* \leq -3$ and $\log_{10}C^* \leq -2$, and $GR_{1.5\text{-}3}$ and $GR_{7\text{-}25}$. Isopleth plots for (a-b) estimated absolute concentrations of OOMs with $\log_{10}C^* \leq -3$ and $\log_{10}C^* \leq -2$ and (c-d) $GR_{1.5\text{-}3}$ and $GR_{7\text{-}25}$ as a function of NO_x and AVOC concentrations. Black filled circles represent the daily average levels of AVOCs and NO_x measured in NPF days in autumn 2018 and 2021 in Beijing. The whiskers correspond to the 25th and 75th percentiles of NO_x and AVOC concentrations.

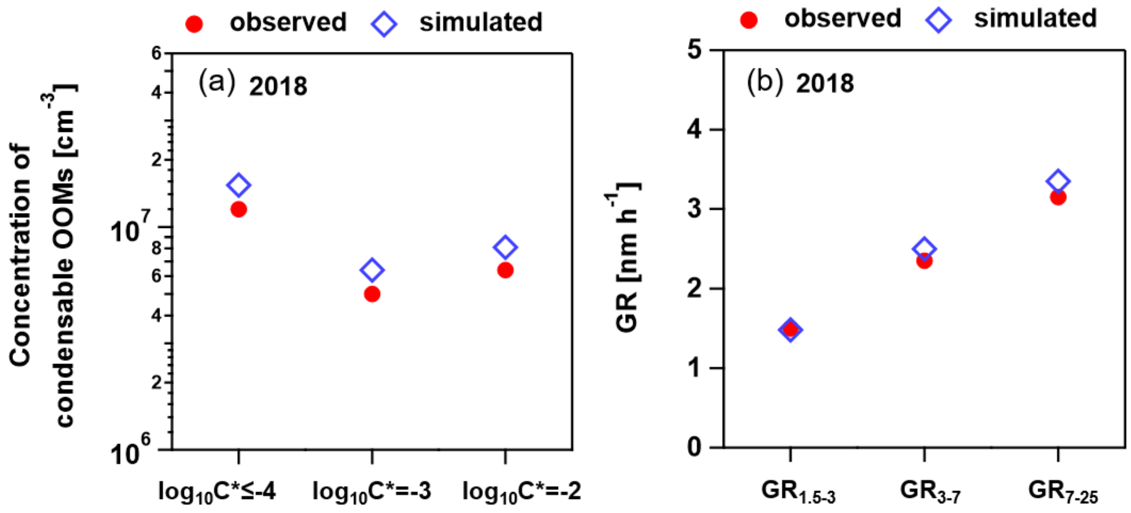

Supplementary Fig. 20. Comparison between simulated and measured OOMs and GR. The simulated and observed (a) concentrations of OOMs with different volatility ranges ($\log_{10}C^* \leq -4$, $\log_{10}C^* = -3$, and $\log_{10}C^* = -2$) and (b) the GR values ($GR_{1.5\text{-}3}$, $GR_{3\text{-}7}$, and $GR_{7\text{-}25}$) in autumn 2018. The observed OOM concentration is from Qiao, et al.[7]. The observed GR is the average value measured in autumn 2018 in this study.

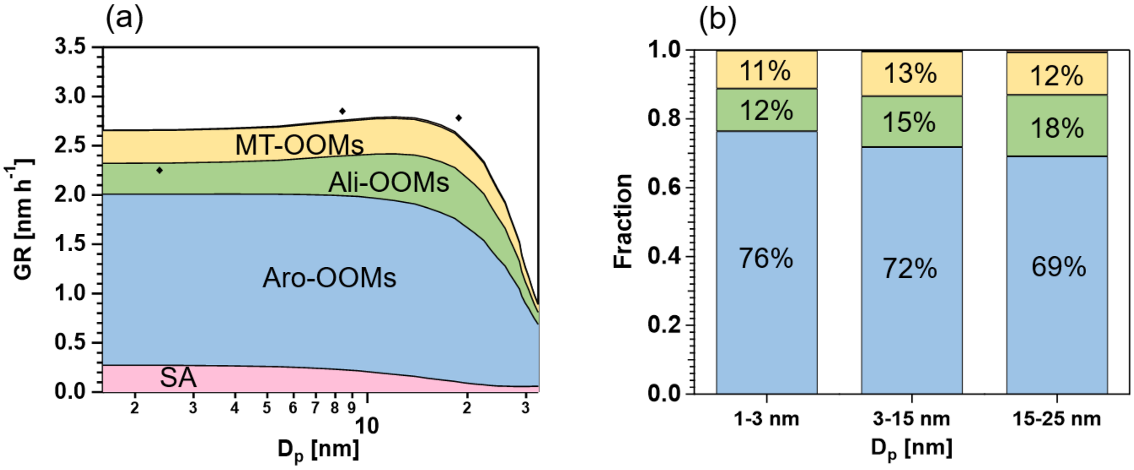

Supplementary Fig. 21. Contribution of OOMs from different sources to GR during the observation in autumn 2021 in Beijing. Contribution of (a) SA and OOMs with different sources to GR as a function of particle size on 19 October 2021. The dots are the observed GR (mode-fitting method). (b) The relative fraction of the GR contributed by the OOMs with different source in different size bins.

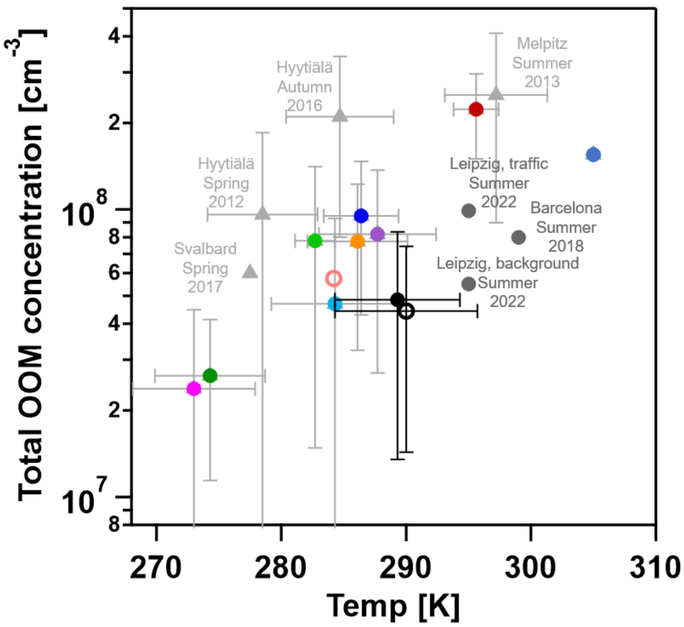

Supplementary Fig. 22. Relationships between total OOM concentrations and temperature from different measurements. The colorful data points are shown in Fig. 3c. Data for spring 2017 in Svalbard are from Beck, et al.[17], data for spring 2012 in Hyytiälä are from Yan, et al.[18], data for autumn 2016 in Hyytiälä are from Zha, et al.[19], data for summer 2013 in Melpitz are from Mutzel, et al.[20], data for summer 2018 in Barcelona are from Brean, et al.[21], data for summer 2022 in Leipzig are from Brean, et al.[22]. The filled circles and hollow circles are average values from whole measurement and NPF days in urban sites, respectively. The triangle are average values from whole measurement in forest and remote sites. The gray error bars show standard deviations. It's found that OOMs in forest and remote environments are, in general, higher than in urban areas. This possibly because the OOM yield of biogenic VOCs is higher than that of anthropogenic VOCs[23,24]. In addition, the observation results in Leipzig during the summer of 2022 indicate that under the similar photochemical reactivity, OOM concentration is greater at the traffic site than urban background site. This also reflects the crucial role of AVOCs in OOM formation.

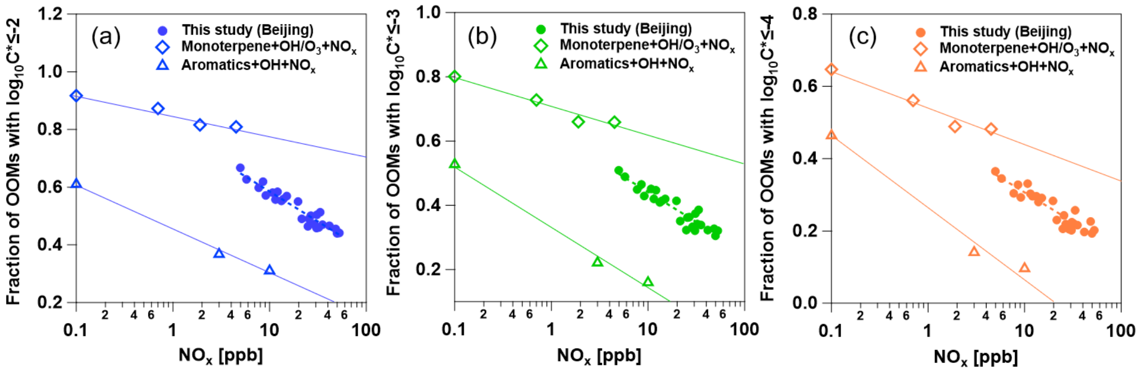

Supplementary Fig. 23. The effect of NO_x on the fraction of condensable OOMs. Relationships between fraction of OOMs with (a) $\log_{10}C^* \leq -2$, (b) $\log_{10}C^* \leq -3$, and (c) $\log_{10}C^* \leq -4$ and NO_x concentrations. The solid circular markers are the results from the observation in autumn 2021 in Beijing. The dashed lines represent the logarithmic fitting result. The quadrilateral and triangular markers are the results from laboratory experiment (Monoterpene + OH/O_3 + NO_x)[25] and model simulation (Aromatics + OH + NO_x)[26], respectively. It should be noted that NO_x with near zero level in laboratory experiment and model simulation was plotted as 0.1 ppb here. The solid lines represent the logarithmic fitting result.

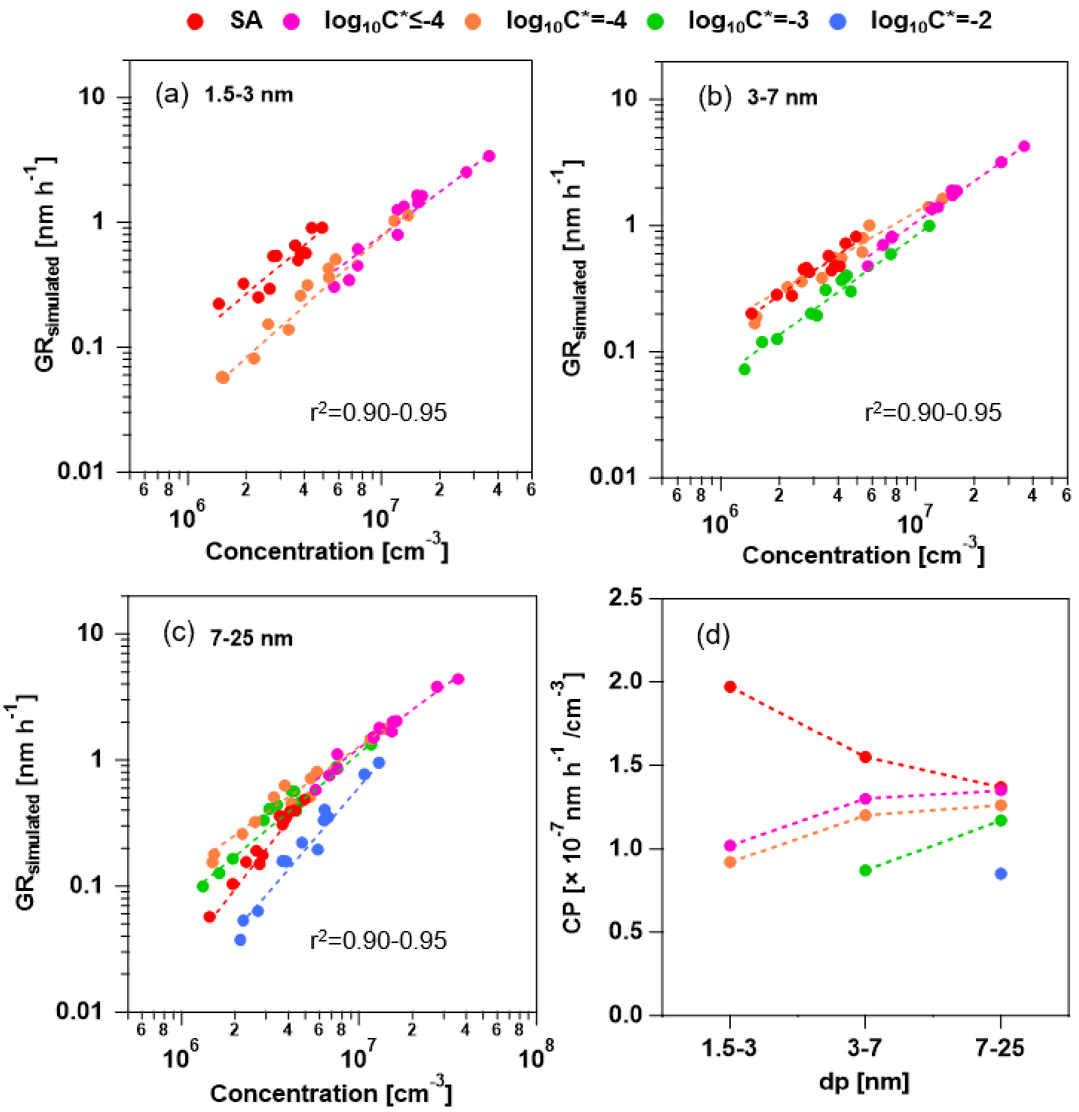

Supplementary Fig. 24. Condensation potential of condensable vapors with different volatility during the observation in autumn 2021 in Beijing based on daily average vapor concentrations. Relationships between simulated condensation GR and daily average vapor concentrations for (a) 1.5-3 nm, (b) 3-7 nm, and (c) 7-25 nm particles. The vapors include sulfuric acid, OOMs with $\log_{10}C^* \leq -4$, $\log_{10}C^* = -4$, -3, and -2. Dashed lines are the linear regressions for different vapors. (d) Slopes of linear regressions ($GR_{simulated}$ vs concentration), i.e., condensation potential (CP), for different vapors in particle size bins.

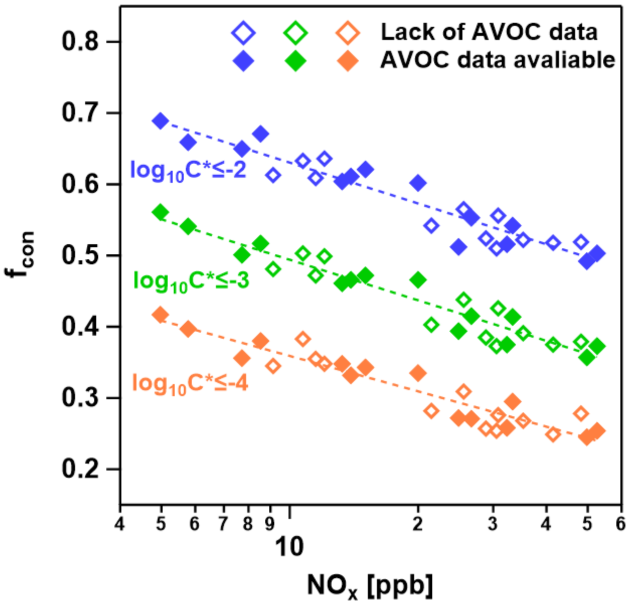

Supplementary Fig. 25. Relationships between daily average fraction of condensable OOMs (f_{con}) and daily average NO_x concentrations at campaign-average temperature (289 K) for days with available AVOC data and lack of AVOC data during the observation in autumn 2021 in Beijing, respectively. The dashed lines represent the logarithmic fitting line of datapoints from all measurement days (the same as Fig. 3b).

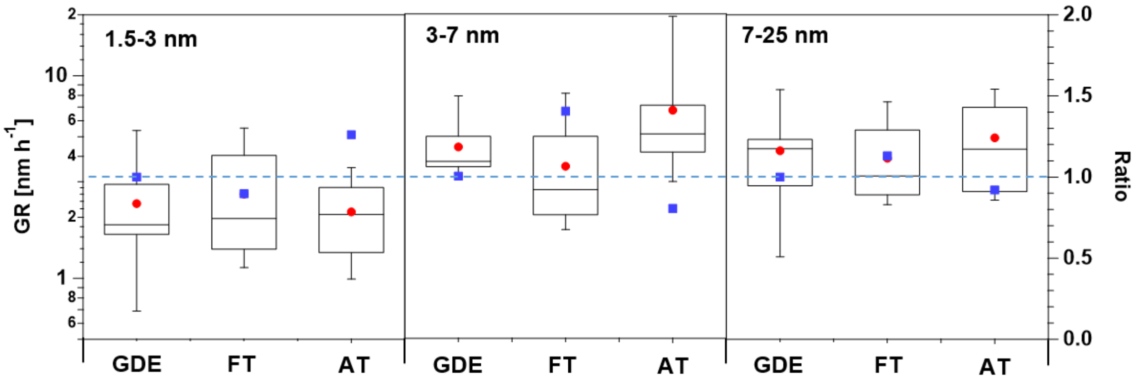

Supplementary Fig. 26. Growth rate (GR) calculated using the aerosol general dynamic equation (GDE) method, mode-fitting (FT) method and appearance time (AT) method in different size bins during the observation in autumn 2021 in Beijing. The whiskers above and below the boxes are the 90th and 10th percentiles; the upper and lower boundaries of the boxes indicate the 75 and 25th percentiles; and the lines and red markers are the median and mean values, respectively. The blue dots are the ratios of GR_{GDE} to GR_{FT} or GR_{AT}.

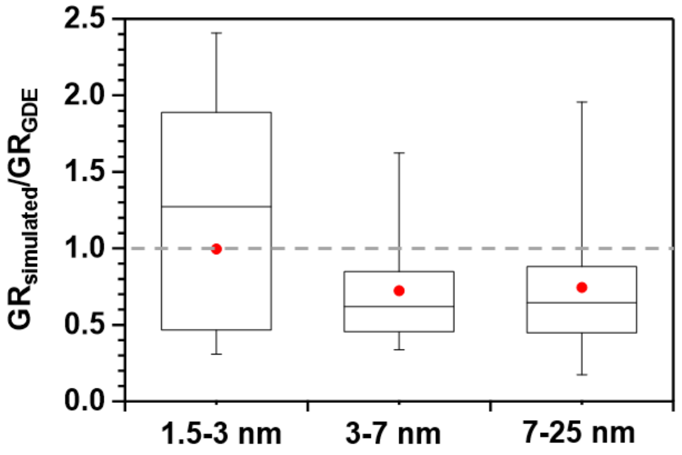

Supplementary Fig. 27. Ratios of simulated GR and GR from GDE method for different particle size bins during the observation in autumn 2021 in Beijing. The whiskers are the 90th and 10th percentiles; the upper and lower boundaries of the boxes indicate the 75 and 25th percentiles; and the lines and red markers are the median and mean values, respectively.

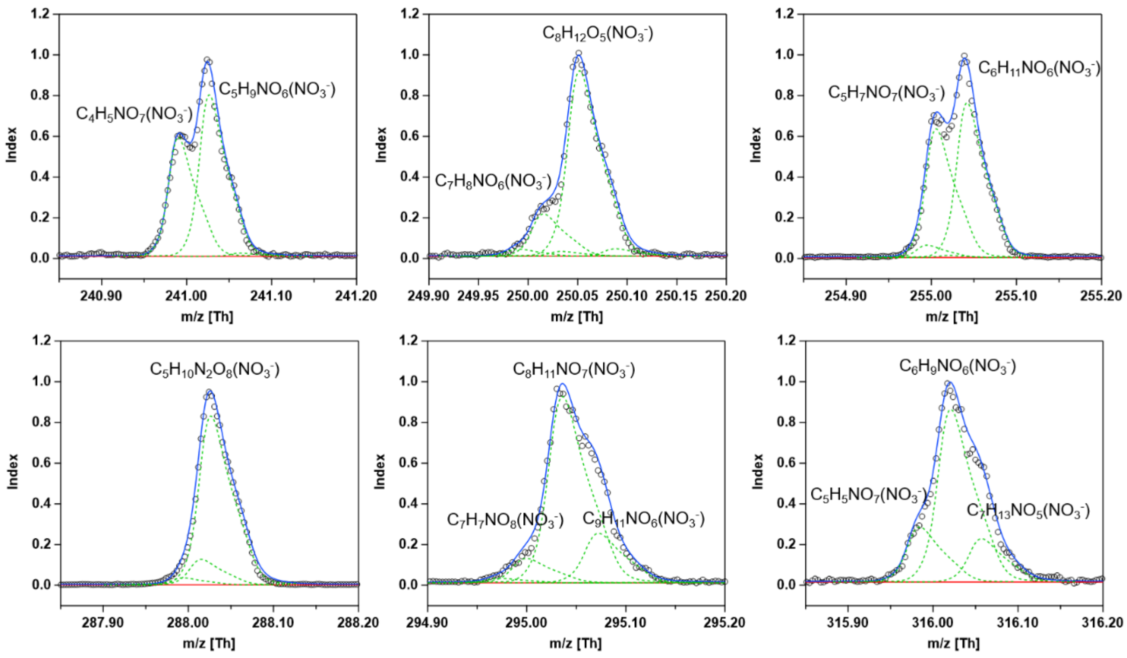

Supplementary Fig. 28. Six examples for peak identification in tofTool. Black circles are the detected signals. Green dotted peaks are the fitted peak. Blue lines are the sum of fitted peaks. Red lines are the residuals.

Supplementary Table 1. The observed $GR_{1.5\text{-}3}$, $GR_{3\text{-}7}$ and $GR_{7\text{-}25}$ using mode-fitting method in autumn 2021.

DATE	$GR_{-1.5\text{-}3}$ (nm h^{-1})	$GR_{3\text{-}7}$ (nm h^{-1})	$GR_{7\text{-}25}$ (nm h^{-1})
20210921	1.5	1.4	2.6
20210929	4.4	7.6	7.4
20211001	1.8	3.2	6.3
20211010	2.1	2.4	2.1
20211011	-	3.6	4.3
20211012	3.0	3.5	5.6
20211015	-	-	-
20211016	2.5	2.1	3.0
20211019	2.3	3.3	3.6
20211021	1.5	2.6	2.7
20211022	2.9	4.4	4.0
20211027	1.3	3.2	3.1
20211031	1.4	2.1	1.8

"-" represent that GR cannot be obtained due to the lack of the data.

Supplementary Table 2. Instruments used in this study and the parameters they measured.

Instrument	Manufacturer (country)	Time resolution	Parameters
Meteorological station	Met one (USA)	1 min	WS, WD, Temp, RH
Model 49i	Thermo (USA)	1 min	O_3
Model 42i	Thermo (USA)	1 min	NO, NO_2
Model 43i-TLE	Thermo (USA)	1 min	SO_2
Spectroradiometer	PKU (CHN)	1 min	$J(O^1D)$
Online GC-MS	PKU (CHN)	1 h	Gaseous VOCs
PSM	Airmodus (Finland)	4 min	PNSD of 1.3-3 nm
Nano-SMPS	TSI (USA)	5 min	PNSD of 3-45 nm particles
Long-SMPS	TSI (USA)	5 min	PNSD of 45-698 nm particles
Nitrate CI-APi-TOF	Aerodyne (USA)	1 s	Sulfuric acid, OOMs

Supplementary Table 3. AVOC species used for the analysis in this study.

Type	Species	nC	Type	Species	nC
	Ethane	2		Benzene	6
	Ethylene	2		Toluene	7
	Propane	3		Ethylbenzene	8
	Propylene	3		m/p-Xylene	8
	Isobutane	4		o-Xylene	8
	Trans-2-butene	4		Styrene	8
Aliphatic	1-Butene	4	Aromatic	iso-Propylbenzene	9
	Cis-2-butene	4		n-Propylbenzene	9
	Cyclopentane	5		m-ethyltoluene	9
	Isopentane	5		p-ethyltoluene	9
	n-Pentane	5		1,3,5-Trimethylbenzene	9
	1-Pentene	5		o-ethyltoluene	9
	trans-2-Pentene	5		1,2,4-Trimethylbenzene	9
	cis-2-Pentene	5		1,2,3-Trimethylbenzene	9
	2,3-Dimethylbutane	6		m-diethylbenzene	10
	2-Methylpentane	6		p-diethylbenzene	10
	3-Methylpentane	6			
	1-Hexene	6			
	n-Hexane	6			
	Methylcyclopentane	6			
	Cyclohexane	6			
	2-Methylhexane	7			
Aliphatic	2,3-Dimethylpentane	7			
	3-Methylhexane	7			
	n-Heptane	7			
	Methylcyclohexane	7			
	2,2,4-Trimethylpentane	8			
	2,3,4-Trimethylpentane	8			
	2-Methylheptane	8			
	3-Methylheptane	8			
	n-octane	8			
	n-Nonane	9			
	n-Decane	10			

Supplementary References

1. Fang, X. *et al.* New particle formation and its CCN enhancement in the Yangtze River Delta under the control of continental and marine air masses. *Atmos. Environ.* **254**, 118400, (2021).
2. Dal Maso, M., Kulmala, M., Riipinen, I. & Wagner, R. Formation and growth of fresh atmospheric aerosols: Eight years of aerosol size distribution data from SMEAR II, Hyytiälä, Finland. *Boreal. Environ. Res.* **10**, 323-336, (2005).
3. Zamora, M. L. *et al.* Wintertime aerosol properties in Beijing. *Atmos. Chem. Phys.* **19**, 14329-14338, (2019).
4. Wang, W. *et al.* The impact of aerosols on photolysis frequencies and ozone production in Beijing during the 4-year period 2012–2015. *Atmos. Chem. Phys.* **19**, 9413-9429, (2019).
5. Fang, X. *et al.* Observational Evidence for the Involvement of Dicarboxylic Acids in Particle Nucleation. *Environ. Sci. Technol. Lett.* **7**, 388-394, (2020).
6. Nie, W. *et al.* Secondary organic aerosol formed by condensing anthropogenic vapours over China's megacities. *Nat. Geosci.* **15**, 255-261, (2022).
7. Qiao, X. *et al.* Contribution of atmospheric oxygenated organic compounds to particle growth in an urban environment. *Environ. Sci. Technol.* **55**, 13646-13656, (2021).
8. Ehn, M. *et al.* A large source of low-volatility secondary organic aerosol. *Nature* **506**, 476-479, (2014).
9. Tröstl, J. *et al.* The role of low-volatility organic compounds in initial particle growth in the atmosphere. *Nature* **533**, 527-531, (2016).
10. Yang, C. *et al.* Molecular composition of anthropogenic oxygenated organic molecules and their contribution to organic aerosol in a coastal city. *Environ. Sci. Technol.* **57**, 15956-15967, (2023).
11. Yu, H. *et al.* Nucleation and growth of sub-3 nm particles in the polluted urban atmosphere of a megacity in China. *Atmos. Chem. Phys.* **16**, 2641-2657, (2016).
12. Kürten, A., Rondo, L., Ehrhart, S. & Curtius, J. Calibration of a chemical ionization mass spectrometer for the measurement of gaseous sulfuric acid. *J. Phys. Chem. A.* **116**, 6375-6386, (2012).
13. Guo, Y. *et al.* Seasonal variation in oxygenated organic molecules in urban Beijing and their contribution to secondary organic aerosol. *Atmos. Chem. Phys.* **22**, 10077-10097, (2022).
14. Tian, L. *et al.* Enigma of urban gaseous oxygenated organic molecules: precursor type, role of NOx, and degree of oxygenation. *Environ. Sci. Technol.* **57**, 64–75, (2022).
15. Zheng, P. *et al.* Molecular characterization of oxygenated organic molecules and their dominating roles in particle growth in Hong Kong. *Environ. Sci. Technol.* **57**, 7764-7776, (2023).
16. Bianchi, F. *et al.* The role of highly oxygenated molecules (HOMs) in determining the composition of ambient ions in the boreal forest. *Atmos. Chem. Phys.* **17**, 13819-13831, (2017).
17. Beck, L. J. *et al.* Differing Mechanisms of New Particle Formation at Two Arctic Sites. *Geophys. Res. Lett.* **48**, e2020GL091334, (2021).

18. Yan, C. *et al.* Source characterization of Highly Oxidized Multifunctional Compounds in a Boreal Forest Environment using Positive Matrix Factorization. *Atmos. Chem. Phys.* **16**, 12715–12731, (2016).
19. Zha, Q. *et al.* Vertical characterization of highly oxygenated molecules (HOMs) below and above a boreal forest canopy. *Atmos. Chem. Phys.* **18**, 17437-17450, (2018).
20. Mutzel, A. *et al.* Highly Oxidized Multifunctional Organic Compounds Observed in Tropospheric Particles: A Field and Laboratory Study. *Environ. Sci. Technol.* **49**, 7754-7761, (2015).
21. Brean, J. *et al.* Molecular insights into new particle formation in Barcelona, Spain. *Atmos. Chem. Phys.* **20**, 10029-10045, (2020).
22. Brean, J. *et al.* Road Traffic Emissions Lead to Much Enhanced New Particle Formation through Increased Growth Rates. *Environ. Sci. Technol.* **58**, 10664-10674, (2024).
23. Berndt, T. *et al.* Hydroxyl radical-induced formation of highly oxidized organic compounds. *Nat. Commun.* **7**, 13677, (2016).
24. Garmash, O. *et al.* Multi-generation OH oxidation as a source for highly oxygenated organic molecules from aromatics. *Atmos. Chem. Phys.* **20**, 515-537, (2020).
25. Yan, C. *et al.* Size-dependent influence of NOx on the growth rates of organic aerosol particles. *Sci. Adv.* **6**, eaay4945, (2020).
26. Yin, D. *et al.* Fostering a holistic understanding of the full volatility spectrum of organic compounds from benzene series precursors through mechanistic modeling. *Environ. Sci. Technol.* **58**, 8380-8392, (2024).
